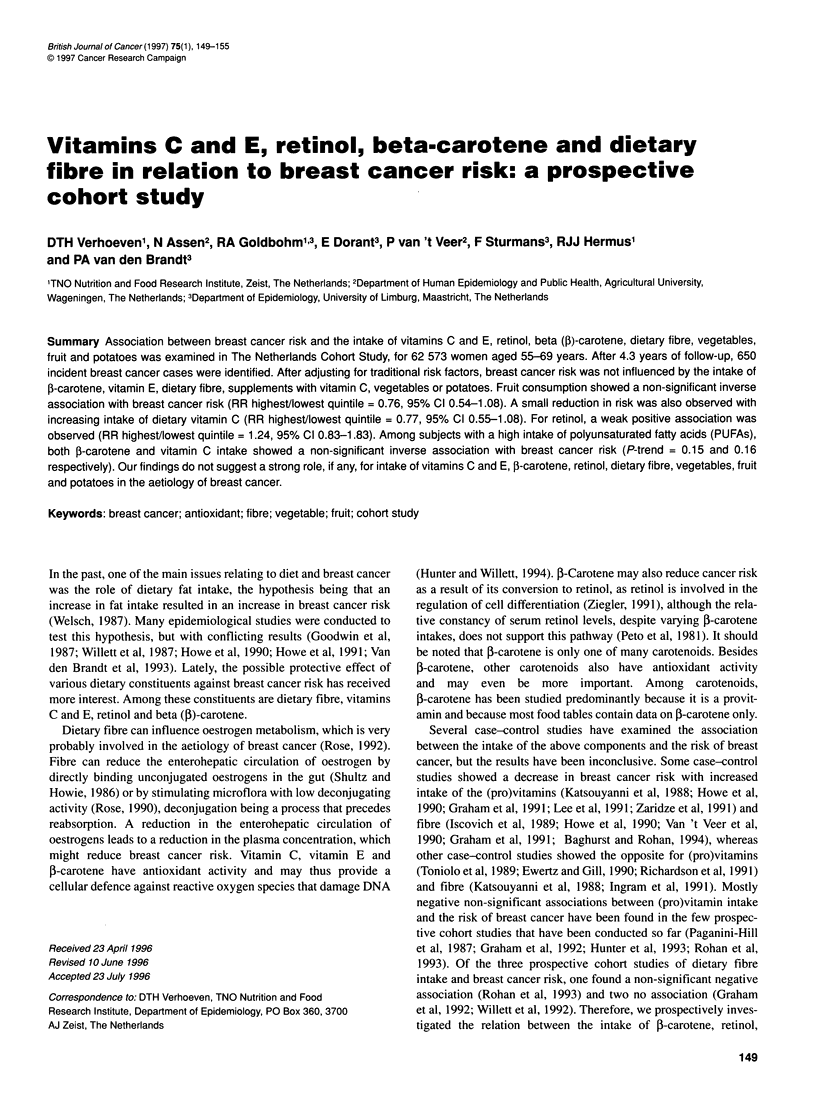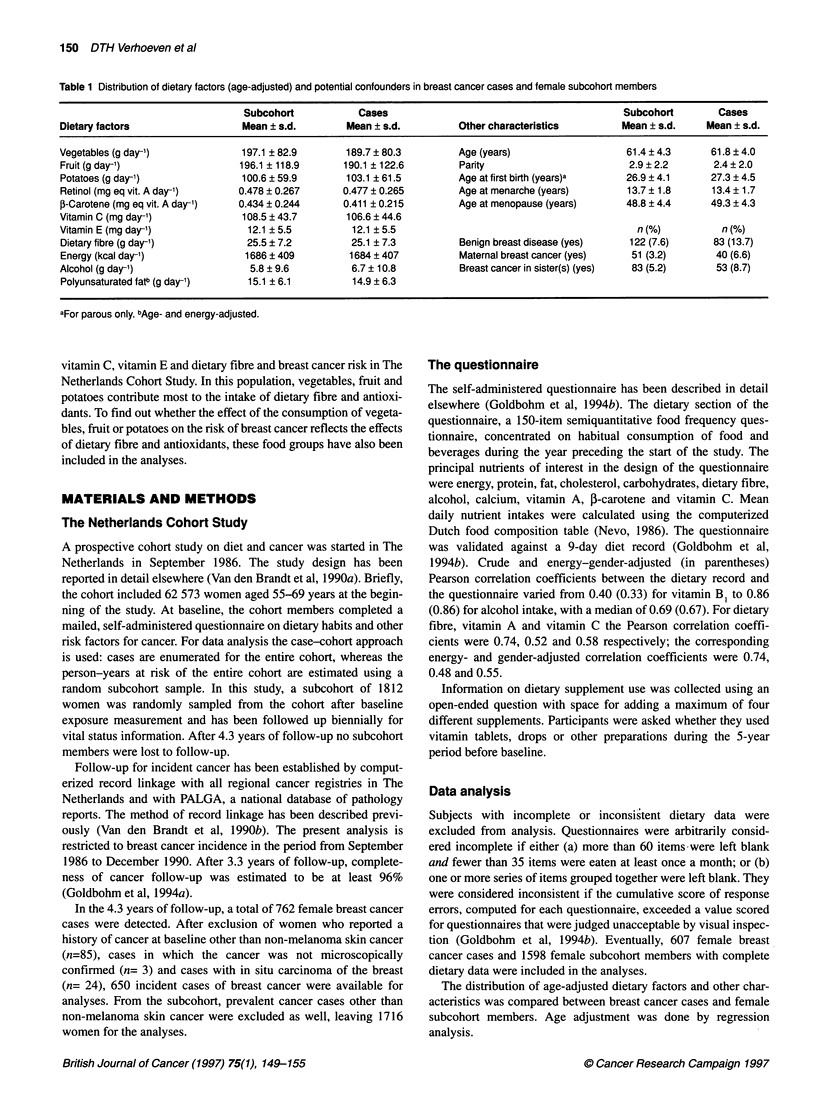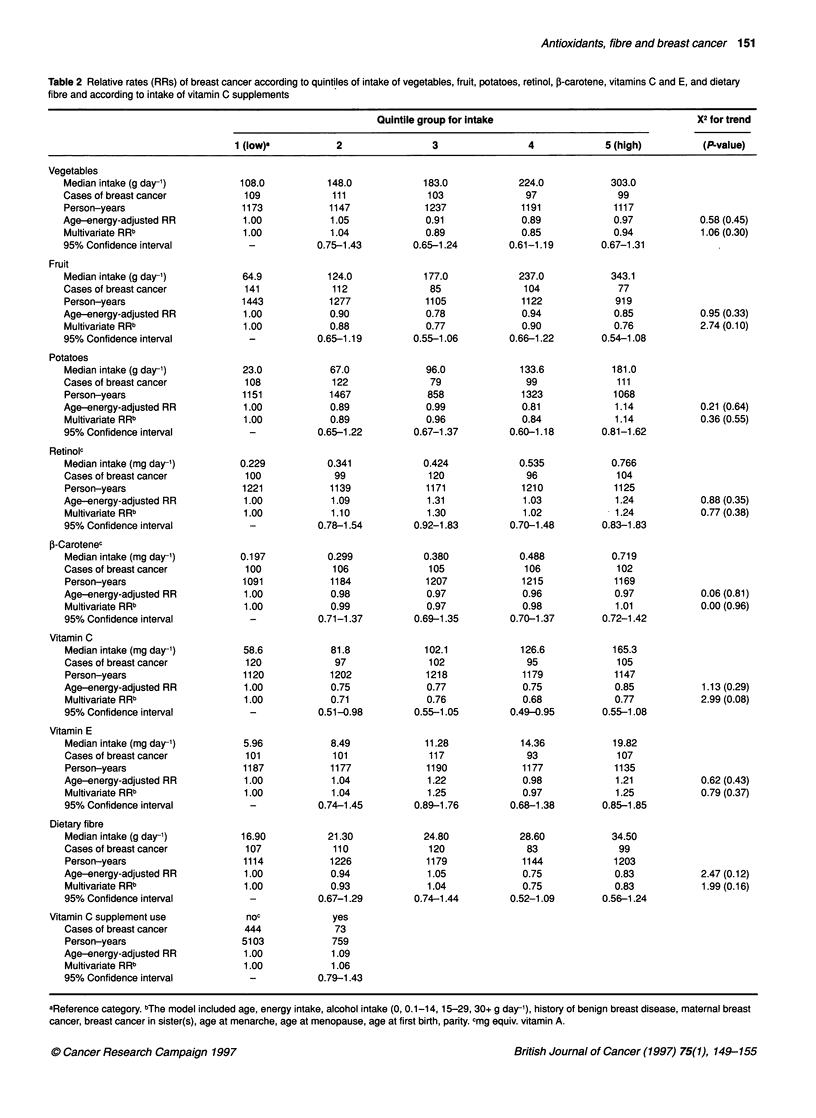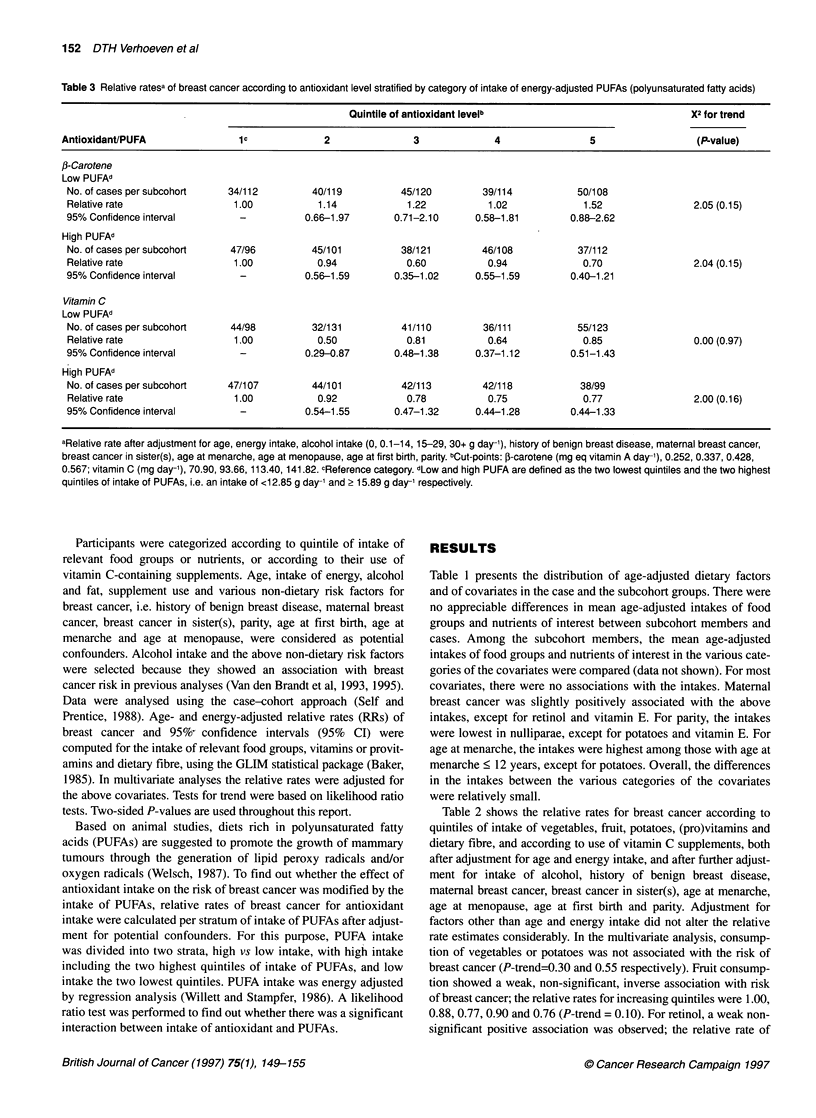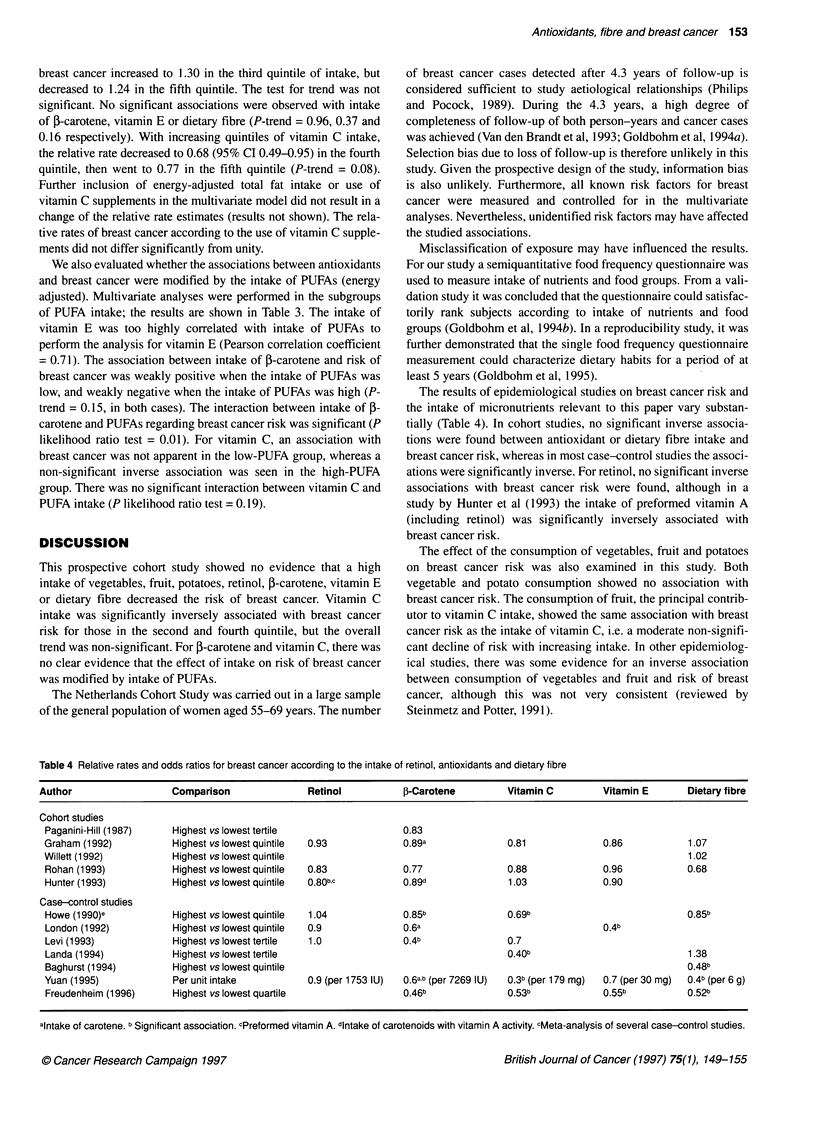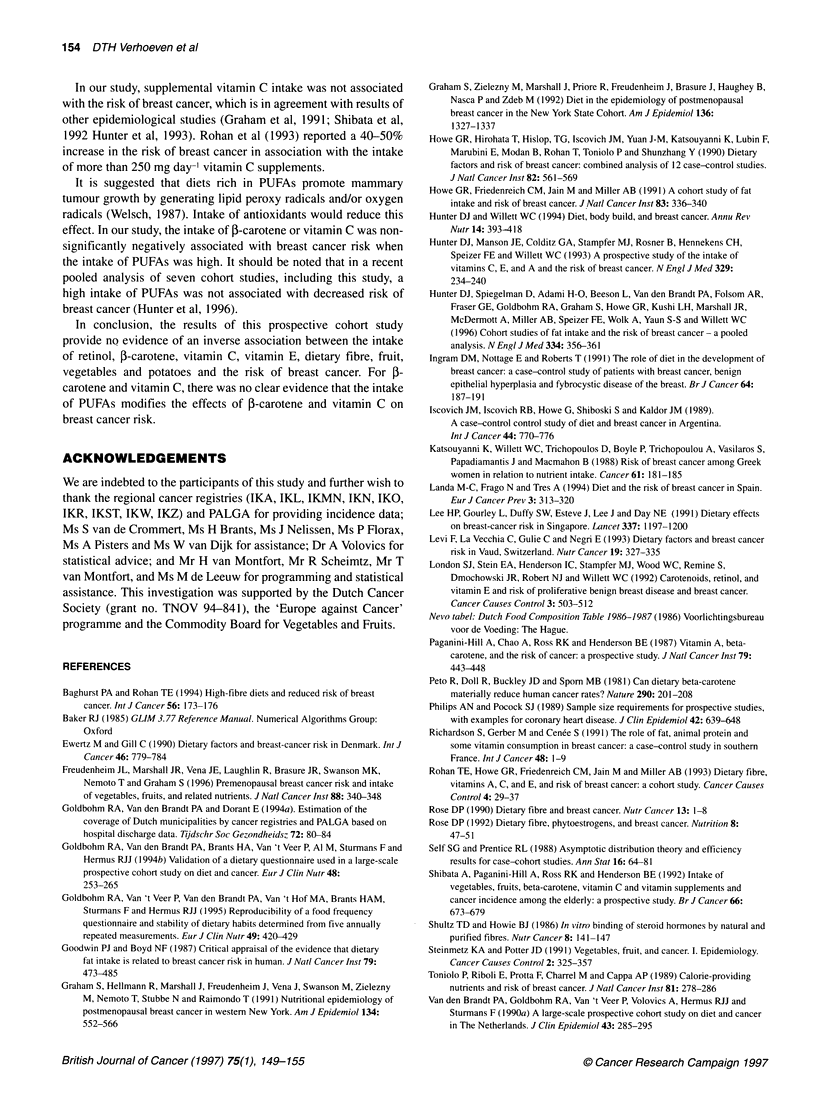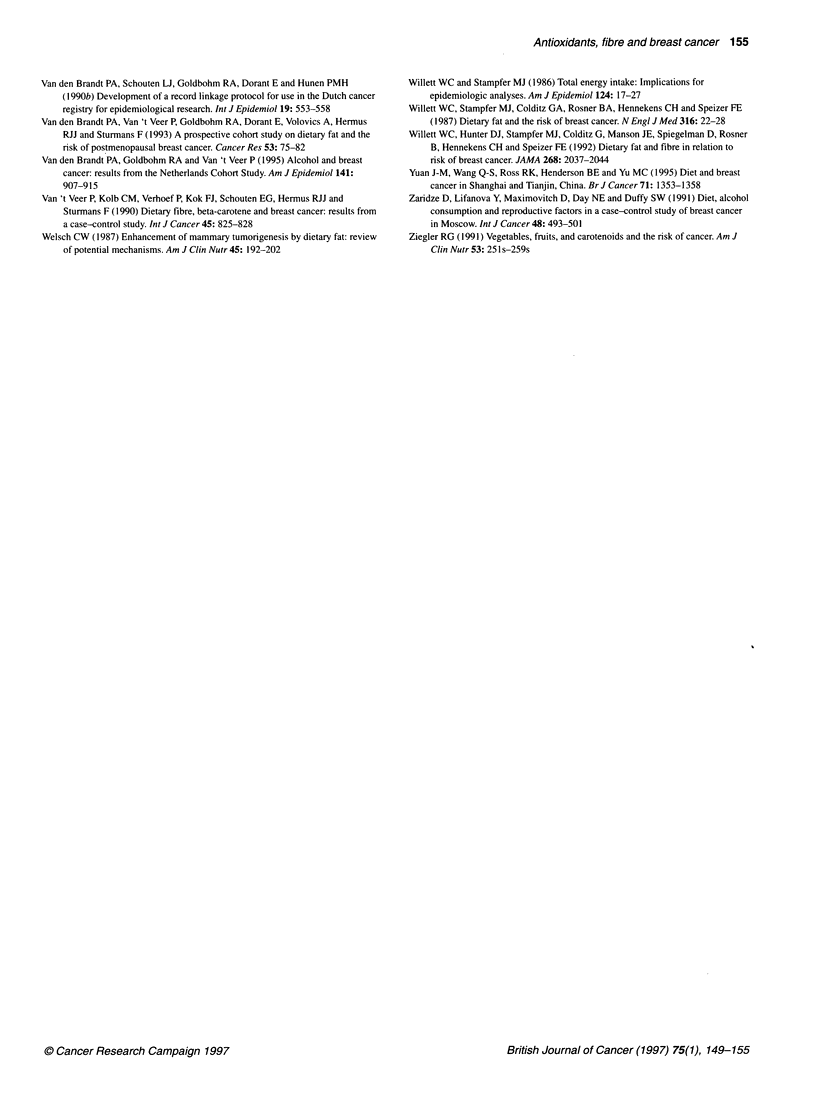# Vitamins C and E, retinol, beta-carotene and dietary fibre in relation to breast cancer risk: a prospective cohort study.

**DOI:** 10.1038/bjc.1997.25

**Published:** 1997

**Authors:** D. T. Verhoeven, N. Assen, R. A. Goldbohm, E. Dorant, P. van 't Veer, F. Sturmans, R. J. Hermus, P. A. van den Brandt

**Affiliations:** TNO Nutrition and Food Research Institute, Zeist, The Netherlands.

## Abstract

Association between breast cancer risk and the intake of vitamins C and E, retinol, beta (beta)-carotene, dietary fibre, vegetables, fruit and potatoes was examined in The Netherlands Cohort Study, for 62,573 women aged 55-69 years. After 4.3 years of follow-up, 650 incident breast cancer cases were identified. After adjusting for traditional risk factors, breast cancer risk was not influenced by the intake of beta-carotene, vitamin E, dietary fibre, supplements with vitamin C, vegetables or potatoes. Fruit consumption showed a non-significant inverse association with breast cancer risk (RR highest/lowest quintile = 0.76, 95% CI 0.54-1.08). A small reduction in risk was also observed with increasing intake of dietary vitamin C (RR highest/lowest quintile = 0.77, 95% CI 0.55-1.08). For retinol, a weak positive association was observed (RR highest/lowest quintile = 1.24, 95% CI 0.83-1.83). Among subjects with a high intake of polyunsaturated fatty acids (PUFAs), both beta-carotene and vitamin C intake showed a non-significant inverse association with breast cancer risk (P-trend = 0.15 and 0.16 respectively). Our findings do not suggest a strong role, if any, for intake of vitamins C and E, beta-carotene, retinol, dietary fibre, vegetables, fruit and potatoes in the aetiology of breast cancer.


British Journal of Cancer (1997) 75(1), 149–155
© 1997 Cancer Research Campaign

### Vitamins C and E, retinol, beta-carotene and dietary fibre in relation to breast cancer risk: a prospective cohort study

**DTH Verhoeven[1], N Assen[2], RA Goldbohm[1,3], E Dorant[3], P van 't Veer[2], F Sturmans[3], RJJ Hermus[1] and PA van den Brandt[3]**

[1]TNO Nutrition and Food Research Institute, Zeist, The Netherlands; [2]Department of Human Epidemiology and Public Health, Agricultural University, Wageningen, The Netherlands; [3]Department of Epidemiology, University of Limburg, Maastricht, The Netherlands

**Summary** Association between breast cancer risk and the intake of vitamins C and E, retinol, beta (β)-carotene, dietary fibre, vegetables, fruit and potatoes was examined in The Netherlands Cohort Study, for 62 573 women aged 55–69 years. After 4.3 years of follow-up, 650 incident breast cancer cases were identified. After adjusting for traditional risk factors, breast cancer risk was not influenced by the intake of β-carotene, vitamin E, dietary fibre, supplements with vitamin C, vegetables or potatoes. Fruit consumption showed a non-significant inverse association with breast cancer risk (RR highest/lowest quintile = 0.76, 95% CI 0.54–1.08). A small reduction in risk was also observed with increasing intake of dietary vitamin C (RR highest/lowest quintile = 0.77, 95% CI 0.55–1.08). For retinol, a weak positive association was observed (RR highest/lowest quintile = 1.24, 95% CI 0.83–1.83). Among subjects with a high intake of polyunsaturated fatty acids (PUFAs), both β-carotene and vitamin C intake showed a non-significant inverse association with breast cancer risk (*P*-trend = 0.15 and 0.16 respectively). Our findings do not suggest a strong role, if any, for intake of vitamins C and E, β-carotene, retinol, dietary fibre, vegetables, fruit and potatoes in the aetiology of breast cancer.

**Keywords**: breast cancer; antioxidant; fibre; vegetable; fruit; cohort study

In the past, one of the main issues relating to diet and breast cancer was the role of dietary fat intake, the hypothesis being that an increase in fat intake resulted in an increase in breast cancer risk (Welsch, 1987). Many epidemiological studies were conducted to test this hypothesis, but with conflicting results (Goodwin et al, 1987; Willett et al, 1987; Howe et al, 1990; Howe et al, 1991; Van den Brandt et al, 1993). Lately, the possible protective effect of various dietary constituents against breast cancer risk has received more interest. Among these constituents are dietary fibre, vitamins C and E, retinol and beta (β)-carotene.

Dietary fibre can influence oestrogen metabolism, which is very probably involved in the aetiology of breast cancer (Rose, 1992). Fibre can reduce the enterohepatic circulation of oestrogen by directly binding unconjugated oestrogens in the gut (Shultz and Howie, 1986) or by stimulating microflora with low deconjugating activity (Rose, 1990), deconjugation being a process that precedes reabsorption. A reduction in the enterohepatic circulation of oestrogens leads to a reduction in the plasma concentration, which might reduce breast cancer risk. Vitamin C, vitamin E and β-carotene have antioxidant activity and may thus provide a cellular defence against reactive oxygen species that damage DNA (Hunter and Willett, 1994). β-Carotene may also reduce cancer risk as a result of its conversion to retinol, as retinol is involved in the regulation of cell differentiation (Ziegler, 1991), although the relative constancy of serum retinol levels, despite varying β-carotene intakes, does not support this pathway (Peto et al, 1981). It should be noted that β-carotene is only one of many carotenoids. Besides β-carotene, other carotenoids also have antioxidant activity and may even be more important. Among carotenoids, β-carotene has been studied predominantly because it is a provitamin and because most food tables contain data on β-carotene only.

Several case–control studies have examined the association between the intake of the above components and the risk of breast cancer, but the results have been inconclusive. Some case–control studies showed a decrease in breast cancer risk with increased intake of the (pro)vitamins (Katsouyanni et al, 1988; Howe et al, 1990; Graham et al, 1991; Lee et al, 1991; Zaridze et al, 1991) and fibre (Iscovich et al, 1989; Howe et al, 1990; Van 't Veer et al, 1990; Graham et al, 1991; Baghurst and Rohan, 1994), whereas other case–control studies showed the opposite for (pro)vitamins (Toniolo et al, 1989; Ewertz and Gill, 1990; Richardson et al, 1991) and fibre (Katsouyanni et al, 1988; Ingram et al, 1991). Mostly negative non-significant associations between (pro)vitamin intake and the risk of breast cancer have been found in the few prospective cohort studies that have been conducted so far (Paganini-Hill et al, 1987; Graham et al, 1992; Hunter et al, 1993; Rohan et al, 1993). Of the three prospective cohort studies of dietary fibre intake and breast cancer risk, one found a non-significant negative association (Rohan et al, 1993) and two no association (Graham et al, 1992; Willett et al, 1992). Therefore, we prospectively investigated the relation between the intake of β-carotene, retinol,

*Received 23 April 1996*
*Revised 10 June 1996*
*Accepted 23 July 1996*

*Correspondence to:* DTH Verhoeven, TNO Nutrition and Food Research Institute, Department of Epidemiology, PO Box 360, 3700 AJ Zeist, The Netherlands

**Table 1** Distribution of dietary factors (age-adjusted) and potential confounders in breast cancer cases and female subcohort members

| Dietary factors | Subcohort Mean ± s.d. | Cases Mean ± s.d. | Other characteristics | Subcohort Mean ± s.d. | Cases Mean ± s.d. |
|---|---|---|---|---|---|
| Vegetables (g day$^{-1}$) | 197.1 ± 82.9 | 189.7 ± 80.3 | Age (years) | 61.4 ± 4.3 | 61.8 ± 4.0 |
| Fruit (g day$^{-1}$) | 196.1 ± 118.9 | 190.1 ± 122.6 | Parity | 2.9 ± 2.2 | 2.4 ± 2.0 |
| Potatoes (g day$^{-1}$) | 100.6 ± 59.9 | 103.1 ± 61.5 | Age at first birth (years)[a] | 26.9 ± 4.1 | 27.3 ± 4.5 |
| Retinol (mg eq vit. A day$^{-1}$) | 0.478 ± 0.267 | 0.477 ± 0.265 | Age at menarche (years) | 13.7 ± 1.8 | 13.4 ± 1.7 |
| β-Carotene (mg eq vit. A day$^{-1}$) | 0.434 ± 0.244 | 0.411 ± 0.215 | Age at menopause (years) | 48.8 ± 4.4 | 49.3 ± 4.3 |
| Vitamin C (mg day$^{-1}$) | 108.5 ± 43.7 | 106.6 ± 44.6 | | | |
| Vitamin E (mg day$^{-1}$) | 12.1 ± 5.5 | 12.1 ± 5.5 | | *n* (%) | *n* (%) |
| Dietary fibre (g day$^{-1}$) | 25.5 ± 7.2 | 25.1 ± 7.3 | Benign breast disease (yes) | 122 (7.6) | 83 (13.7) |
| Energy (kcal day$^{-1}$) | 1686 ± 409 | 1684 ± 407 | Maternal breast cancer (yes) | 51 (3.2) | 40 (6.6) |
| Alcohol (g day$^{-1}$) | 5.8 ± 9.6 | 6.7 ± 10.8 | Breast cancer in sister(s) (yes) | 83 (5.2) | 53 (8.7) |
| Polyunsaturated fat[b] (g day$^{-1}$) | 15.1 ± 6.1 | 14.9 ± 6.3 | | | |

[a]For parous only. [b]Age- and energy-adjusted.

vitamin C, vitamin E and dietary fibre and breast cancer risk in The Netherlands Cohort Study. In this population, vegetables, fruit and potatoes contribute most to the intake of dietary fibre and antioxidants. To find out whether the effect of the consumption of vegetables, fruit or potatoes on the risk of breast cancer reflects the effects of dietary fibre and antioxidants, these food groups have also been included in the analyses.

### MATERIALS AND METHODS

### The Netherlands Cohort Study

A prospective cohort study on diet and cancer was started in The Netherlands in September 1986. The study design has been reported in detail elsewhere (Van den Brandt et al, 1990*a*). Briefly, the cohort included 62 573 women aged 55–69 years at the beginning of the study. At baseline, the cohort members completed a mailed, self-administered questionnaire on dietary habits and other risk factors for cancer. For data analysis the case–cohort approach is used: cases are enumerated for the entire cohort, whereas the person–years at risk of the entire cohort are estimated using a random subcohort sample. In this study, a subcohort of 1812 women was randomly sampled from the cohort after baseline exposure measurement and has been followed up biennially for vital status information. After 4.3 years of follow-up no subcohort members were lost to follow-up.

Follow-up for incident cancer has been established by computerized record linkage with all regional cancer registries in The Netherlands and with PALGA, a national database of pathology reports. The method of record linkage has been described previously (Van den Brandt et al, 1990*b*). The present analysis is restricted to breast cancer incidence in the period from September 1986 to December 1990. After 3.3 years of follow-up, completeness of cancer follow-up was estimated to be at least 96% (Goldbohm et al, 1994*a*).

In the 4.3 years of follow-up, a total of 762 female breast cancer cases were detected. After exclusion of women who reported a history of cancer at baseline other than non-melanoma skin cancer ($n$=85), cases in which the cancer was not microscopically confirmed ($n$= 3) and cases with in situ carcinoma of the breast ($n$= 24), 650 incident cases of breast cancer were available for analyses. From the subcohort, prevalent cancer cases other than non-melanoma skin cancer were excluded as well, leaving 1716 women for the analyses.

### The questionnaire

The self-administered questionnaire has been described in detail elsewhere (Goldbohm et al, 1994*b*). The dietary section of the questionnaire, a 150-item semiquantitative food frequency questionnaire, concentrated on habitual consumption of food and beverages during the year preceding the start of the study. The principal nutrients of interest in the design of the questionnaire were energy, protein, fat, cholesterol, carbohydrates, dietary fibre, alcohol, calcium, vitamin A, β-carotene and vitamin C. Mean daily nutrient intakes were calculated using the computerized Dutch food composition table (Nevo, 1986). The questionnaire was validated against a 9-day diet record (Goldbohm et al, 1994*b*). Crude and energy–gender-adjusted (in parentheses) Pearson correlation coefficients between the dietary record and the questionnaire varied from 0.40 (0.33) for vitamin $B_1$ to 0.86 (0.86) for alcohol intake, with a median of 0.69 (0.67). For dietary fibre, vitamin A and vitamin C the Pearson correlation coefficients were 0.74, 0.52 and 0.58 respectively; the corresponding energy- and gender-adjusted correlation coefficients were 0.74, 0.48 and 0.55.

Information on dietary supplement use was collected using an open-ended question with space for adding a maximum of four different supplements. Participants were asked whether they used vitamin tablets, drops or other preparations during the 5-year period before baseline.

### Data analysis

Subjects with incomplete or inconsistent dietary data were excluded from analysis. Questionnaires were arbitrarily considered incomplete if either (a) more than 60 items were left blank *and* fewer than 35 items were eaten at least once a month; or (b) one or more series of items grouped together were left blank. They were considered inconsistent if the cumulative score of response errors, computed for each questionnaire, exceeded a value scored for questionnaires that were judged unacceptable by visual inspection (Goldbohm et al, 1994*b*). Eventually, 607 female breast cancer cases and 1598 female subcohort members with complete dietary data were included in the analyses.

The distribution of age-adjusted dietary factors and other characteristics was compared between breast cancer cases and female subcohort members. Age adjustment was done by regression analysis.

© Cancer Research Campaign 1997

**Table 2** Relative rates (RRs) of breast cancer according to quintiles of intake of vegetables, fruit, potatoes, retinol, β-carotene, vitamins C and E, and dietary fibre and according to intake of vitamin C supplements

| | Quintile group for intake | | | | | $X^2$ for trend |
|---|---|---|---|---|---|---|
| | 1 (low)[a] | 2 | 3 | 4 | 5 (high) | (*P*-value) |
| Vegetables | | | | | | |
| Median intake (g day$^{-1}$) | 108.0 | 148.0 | 183.0 | 224.0 | 303.0 | |
| Cases of breast cancer | 109 | 111 | 103 | 97 | 99 | |
| Person–years | 1173 | 1147 | 1237 | 1191 | 1117 | |
| Age–energy-adjusted RR | 1.00 | 1.05 | 0.91 | 0.89 | 0.97 | 0.58 (0.45) |
| Multivariate RR[b] | 1.00 | 1.04 | 0.89 | 0.85 | 0.94 | 1.06 (0.30) |
| 95% Confidence interval | – | 0.75–1.43 | 0.65–1.24 | 0.61–1.19 | 0.67–1.31 | |
| Fruit | | | | | | |
| Median intake (g day$^{-1}$) | 64.9 | 124.0 | 177.0 | 237.0 | 343.1 | |
| Cases of breast cancer | 141 | 112 | 85 | 104 | 77 | |
| Person–years | 1443 | 1277 | 1105 | 1122 | 919 | |
| Age–energy-adjusted RR | 1.00 | 0.90 | 0.78 | 0.94 | 0.85 | 0.95 (0.33) |
| Multivariate RR[b] | 1.00 | 0.88 | 0.77 | 0.90 | 0.76 | 2.74 (0.10) |
| 95% Confidence interval | – | 0.65–1.19 | 0.55–1.06 | 0.66–1.22 | 0.54–1.08 | |
| Potatoes | | | | | | |
| Median intake (g day$^{-1}$) | 23.0 | 67.0 | 96.0 | 133.6 | 181.0 | |
| Cases of breast cancer | 108 | 122 | 79 | 99 | 111 | |
| Person–years | 1151 | 1467 | 858 | 1323 | 1068 | |
| Age–energy-adjusted RR | 1.00 | 0.89 | 0.99 | 0.81 | 1.14 | 0.21 (0.64) |
| Multivariate RR[b] | 1.00 | 0.89 | 0.96 | 0.84 | 1.14 | 0.36 (0.55) |
| 95% Confidence interval | – | 0.65–1.22 | 0.67–1.37 | 0.60–1.18 | 0.81–1.62 | |
| Retinol[c] | | | | | | |
| Median intake (mg day$^{-1}$) | 0.229 | 0.341 | 0.424 | 0.535 | 0.766 | |
| Cases of breast cancer | 100 | 99 | 120 | 96 | 104 | |
| Person–years | 1221 | 1139 | 1171 | 1210 | 1125 | |
| Age–energy-adjusted RR | 1.00 | 1.09 | 1.31 | 1.03 | 1.24 | 0.88 (0.35) |
| Multivariate RR[b] | 1.00 | 1.10 | 1.30 | 1.02 | 1.24 | 0.77 (0.38) |
| 95% Confidence interval | – | 0.78–1.54 | 0.92–1.83 | 0.70–1.48 | 0.83–1.83 | |
| β-Carotene[c] | | | | | | |
| Median intake (mg day$^{-1}$) | 0.197 | 0.299 | 0.380 | 0.488 | 0.719 | |
| Cases of breast cancer | 100 | 106 | 105 | 106 | 102 | |
| Person–years | 1091 | 1184 | 1207 | 1215 | 1169 | |
| Age–energy-adjusted RR | 1.00 | 0.98 | 0.97 | 0.96 | 0.97 | 0.06 (0.81) |
| Multivariate RR[b] | 1.00 | 0.99 | 0.97 | 0.98 | 1.01 | 0.00 (0.96) |
| 95% Confidence interval | – | 0.71–1.37 | 0.69–1.35 | 0.70–1.37 | 0.72–1.42 | |
| Vitamin C | | | | | | |
| Median intake (mg day$^{-1}$) | 58.6 | 81.8 | 102.1 | 126.6 | 165.3 | |
| Cases of breast cancer | 120 | 97 | 102 | 95 | 105 | |
| Person–years | 1120 | 1202 | 1218 | 1179 | 1147 | |
| Age–energy-adjusted RR | 1.00 | 0.75 | 0.77 | 0.75 | 0.85 | 1.13 (0.29) |
| Multivariate RR[b] | 1.00 | 0.71 | 0.76 | 0.68 | 0.77 | 2.99 (0.08) |
| 95% Confidence interval | – | 0.51–0.98 | 0.55–1.05 | 0.49–0.95 | 0.55–1.08 | |
| Vitamin E | | | | | | |
| Median intake (mg day$^{-1}$) | 5.96 | 8.49 | 11.28 | 14.36 | 19.82 | |
| Cases of breast cancer | 101 | 101 | 117 | 93 | 107 | |
| Person–years | 1187 | 1177 | 1190 | 1177 | 1135 | |
| Age–energy-adjusted RR | 1.00 | 1.04 | 1.22 | 0.98 | 1.21 | 0.62 (0.43) |
| Multivariate RR[b] | 1.00 | 1.04 | 1.25 | 0.97 | 1.25 | 0.79 (0.37) |
| 95% Confidence interval | – | 0.74–1.45 | 0.89–1.76 | 0.68–1.38 | 0.85–1.85 | |
| Dietary fibre | | | | | | |
| Median intake (g day$^{-1}$) | 16.90 | 21.30 | 24.80 | 28.60 | 34.50 | |
| Cases of breast cancer | 107 | 110 | 120 | 83 | 99 | |
| Person–years | 1114 | 1226 | 1179 | 1144 | 1203 | |
| Age–energy-adjusted RR | 1.00 | 0.94 | 1.05 | 0.75 | 0.83 | 2.47 (0.12) |
| Multivariate RR[b] | 1.00 | 0.93 | 1.04 | 0.75 | 0.83 | 1.99 (0.16) |
| 95% Confidence interval | – | 0.67–1.29 | 0.74–1.44 | 0.52–1.09 | 0.56–1.24 | |
| Vitamin C supplement use | no[c] | yes | | | | |
| Cases of breast cancer | 444 | 73 | | | | |
| Person–years | 5103 | 759 | | | | |
| Age–energy-adjusted RR | 1.00 | 1.09 | | | | |
| Multivariate RR[b] | 1.00 | 1.06 | | | | |
| 95% Confidence interval | – | 0.79–1.43 | | | | |

[a]Reference category. [b]The model included age, energy intake, alcohol intake (0, 0.1–14, 15–29, 30+ g day$^{-1}$), history of benign breast disease, maternal breast cancer, breast cancer in sister(s), age at menarche, age at menopause, age at first birth, parity. [c]mg equiv. vitamin A.

© *Cancer Research Campaign 1997*

**Table 3** Relative rates[a] of breast cancer according to antioxidant level stratified by category of intake of energy-adjusted PUFAs (polyunsaturated fatty acids)

| | Quintile of antioxidant level[b] | | | | | $X^2$ for trend |
|---|---|---|---|---|---|---|
| Antioxidant/PUFA | 1[c] | 2 | 3 | 4 | 5 | (*P*-value) |
| *β-Carotene* | | | | | | |
| Low PUFA[d] | | | | | | |
| No. of cases per subcohort | 34/112 | 40/119 | 45/120 | 39/114 | 50/108 | |
| Relative rate | 1.00 | 1.14 | 1.22 | 1.02 | 1.52 | 2.05 (0.15) |
| 95% Confidence interval | – | 0.66–1.97 | 0.71–2.10 | 0.58–1.81 | 0.88–2.62 | |
| High PUFA[d] | | | | | | |
| No. of cases per subcohort | 47/96 | 45/101 | 38/121 | 46/108 | 37/112 | |
| Relative rate | 1.00 | 0.94 | 0.60 | 0.94 | 0.70 | 2.04 (0.15) |
| 95% Confidence interval | – | 0.56–1.59 | 0.35–1.02 | 0.55–1.59 | 0.40–1.21 | |
| *Vitamin C* | | | | | | |
| Low PUFA[d] | | | | | | |
| No. of cases per subcohort | 44/98 | 32/131 | 41/110 | 36/111 | 55/123 | |
| Relative rate | 1.00 | 0.50 | 0.81 | 0.64 | 0.85 | 0.00 (0.97) |
| 95% Confidence interval | – | 0.29–0.87 | 0.48–1.38 | 0.37–1.12 | 0.51–1.43 | |
| High PUFA[d] | | | | | | |
| No. of cases per subcohort | 47/107 | 44/101 | 42/113 | 42/118 | 38/99 | |
| Relative rate | 1.00 | 0.92 | 0.78 | 0.75 | 0.77 | 2.00 (0.16) |
| 95% Confidence interval | – | 0.54–1.55 | 0.47–1.32 | 0.44–1.28 | 0.44–1.33 | |

[a]Relative rate after adjustment for age, energy intake, alcohol intake (0, 0.1–14, 15–29, 30+ g day$^{-1}$), history of benign breast disease, maternal breast cancer, breast cancer in sister(s), age at menarche, age at menopause, age at first birth, parity. [b]Cut-points: β-carotene (mg eq vitamin A day$^{-1}$), 0.252, 0.337, 0.428, 0.567; vitamin C (mg day$^{-1}$), 70.90, 93.66, 113.40, 141.82. [c]Reference category. [d]Low and high PUFA are defined as the two lowest quintiles and the two highest quintiles of intake of PUFAs, i.e. an intake of <12.85 g day$^{-1}$ and ≥ 15.89 g day$^{-1}$ respectively.

Participants were categorized according to quintile of intake of relevant food groups or nutrients, or according to their use of vitamin C-containing supplements. Age, intake of energy, alcohol and fat, supplement use and various non-dietary risk factors for breast cancer, i.e. history of benign breast disease, maternal breast cancer, breast cancer in sister(s), parity, age at first birth, age at menarche and age at menopause, were considered as potential confounders. Alcohol intake and the above non-dietary risk factors were selected because they showed an association with breast cancer risk in previous analyses (Van den Brandt et al, 1993, 1995). Data were analysed using the case–cohort approach (Self and Prentice, 1988). Age- and energy-adjusted relative rates (RRs) of breast cancer and 95% confidence intervals (95% CI) were computed for the intake of relevant food groups, vitamins or provitamins and dietary fibre, using the GLIM statistical package (Baker, 1985). In multivariate analyses the relative rates were adjusted for the above covariates. Tests for trend were based on likelihood ratio tests. Two-sided *P*-values are used throughout this report.

Based on animal studies, diets rich in polyunsaturated fatty acids (PUFAs) are suggested to promote the growth of mammary tumours through the generation of lipid peroxy radicals and/or oxygen radicals (Welsch, 1987). To find out whether the effect of antioxidant intake on the risk of breast cancer was modified by the intake of PUFAs, relative rates of breast cancer for antioxidant intake were calculated per stratum of intake of PUFAs after adjustment for potential confounders. For this purpose, PUFA intake was divided into two strata, high *vs* low intake, with high intake including the two highest quintiles of intake of PUFAs, and low intake the two lowest quintiles. PUFA intake was energy adjusted by regression analysis (Willett and Stampfer, 1986). A likelihood ratio test was performed to find out whether there was a significant interaction between intake of antioxidant and PUFAs.

### RESULTS

Table 1 presents the distribution of age-adjusted dietary factors and of covariates in the case and the subcohort groups. There were no appreciable differences in mean age-adjusted intakes of food groups and nutrients of interest between subcohort members and cases. Among the subcohort members, the mean age-adjusted intakes of food groups and nutrients of interest in the various categories of the covariates were compared (data not shown). For most covariates, there were no associations with the intakes. Maternal breast cancer was slightly positively associated with the above intakes, except for retinol and vitamin E. For parity, the intakes were lowest in nulliparae, except for potatoes and vitamin E. For age at menarche, the intakes were highest among those with age at menarche ≤ 12 years, except for potatoes. Overall, the differences in the intakes between the various categories of the covariates were relatively small.

Table 2 shows the relative rates for breast cancer according to quintiles of intake of vegetables, fruit, potatoes, (pro)vitamins and dietary fibre, and according to use of vitamin C supplements, both after adjustment for age and energy intake, and after further adjustment for intake of alcohol, history of benign breast disease, maternal breast cancer, breast cancer in sister(s), age at menarche, age at menopause, age at first birth and parity. Adjustment for factors other than age and energy intake did not alter the relative rate estimates considerably. In the multivariate analysis, consumption of vegetables or potatoes was not associated with the risk of breast cancer (*P*-trend=0.30 and 0.55 respectively). Fruit consumption showed a weak, non-significant, inverse association with risk of breast cancer; the relative rates for increasing quintiles were 1.00, 0.88, 0.77, 0.90 and 0.76 (*P*-trend = 0.10). For retinol, a weak non-significant positive association was observed; the relative rate of

 © Cancer Research Campaign 1997

breast cancer increased to 1.30 in the third quintile of intake, but decreased to 1.24 in the fifth quintile. The test for trend was not significant. No significant associations were observed with intake of β-carotene, vitamin E or dietary fibre (*P*-trend = 0.96, 0.37 and 0.16 respectively). With increasing quintiles of vitamin C intake, the relative rate decreased to 0.68 (95% CI 0.49–0.95) in the fourth quintile, then went to 0.77 in the fifth quintile (*P*-trend = 0.08). Further inclusion of energy-adjusted total fat intake or use of vitamin C supplements in the multivariate model did not result in a change of the relative rate estimates (results not shown). The relative rates of breast cancer according to the use of vitamin C supplements did not differ significantly from unity.

We also evaluated whether the associations between antioxidants and breast cancer were modified by the intake of PUFAs (energy adjusted). Multivariate analyses were performed in the subgroups of PUFA intake; the results are shown in Table 3. The intake of vitamin E was too highly correlated with intake of PUFAs to perform the analysis for vitamin E (Pearson correlation coefficient = 0.71). The association between intake of β-carotene and risk of breast cancer was weakly positive when the intake of PUFAs was low, and weakly negative when the intake of PUFAs was high (*P*-trend = 0.15, in both cases). The interaction between intake of β-carotene and PUFAs regarding breast cancer risk was significant (*P* likelihood ratio test = 0.01). For vitamin C, an association with breast cancer was not apparent in the low-PUFA group, whereas a non-significant inverse association was seen in the high-PUFA group. There was no significant interaction between vitamin C and PUFA intake (*P* likelihood ratio test = 0.19).

### DISCUSSION

This prospective cohort study showed no evidence that a high intake of vegetables, fruit, potatoes, retinol, β-carotene, vitamin E or dietary fibre decreased the risk of breast cancer. Vitamin C intake was significantly inversely associated with breast cancer risk for those in the second and fourth quintile, but the overall trend was non-significant. For β-carotene and vitamin C, there was no clear evidence that the effect of intake on risk of breast cancer was modified by intake of PUFAs.

The Netherlands Cohort Study was carried out in a large sample of the general population of women aged 55–69 years. The number of breast cancer cases detected after 4.3 years of follow-up is considered sufficient to study aetiological relationships (Philips and Pocock, 1989). During the 4.3 years, a high degree of completeness of follow-up of both person–years and cancer cases was achieved (Van den Brandt et al, 1993; Goldbohm et al, 1994*a*). Selection bias due to loss of follow-up is therefore unlikely in this study. Given the prospective design of the study, information bias is also unlikely. Furthermore, all known risk factors for breast cancer were measured and controlled for in the multivariate analyses. Nevertheless, unidentified risk factors may have affected the studied associations.

Misclassification of exposure may have influenced the results. For our study a semiquantitative food frequency questionnaire was used to measure intake of nutrients and food groups. From a validation study it was concluded that the questionnaire could satisfactorily rank subjects according to intake of nutrients and food groups (Goldbohm et al, 1994*b*). In a reproducibility study, it was further demonstrated that the single food frequency questionnaire measurement could characterize dietary habits for a period of at least 5 years (Goldbohm et al, 1995).

The results of epidemiological studies on breast cancer risk and the intake of micronutrients relevant to this paper vary substantially (Table 4). In cohort studies, no significant inverse associations were found between antioxidant or dietary fibre intake and breast cancer risk, whereas in most case–control studies the associations were significantly inverse. For retinol, no significant inverse associations with breast cancer risk were found, although in a study by Hunter et al (1993) the intake of preformed vitamin A (including retinol) was significantly inversely associated with breast cancer risk.

The effect of the consumption of vegetables, fruit and potatoes on breast cancer risk was also examined in this study. Both vegetable and potato consumption showed no association with breast cancer risk. The consumption of fruit, the principal contributor to vitamin C intake, showed the same association with breast cancer risk as the intake of vitamin C, i.e. a moderate non-significant decline of risk with increasing intake. In other epidemiological studies, there was some evidence for an inverse association between consumption of vegetables and fruit and risk of breast cancer, although this was not very consistent (reviewed by Steinmetz and Potter, 1991).

**Table 4** Relative rates and odds ratios for breast cancer according to the intake of retinol, antioxidants and dietary fibre

| Author | Comparison | Retinol | β-Carotene | Vitamin C | Vitamin E | Dietary fibre |
|---|---|---|---|---|---|---|
| Cohort studies | | | | | | |
| Paganini-Hill (1987) | Highest *vs* lowest tertile | | 0.83 | | | |
| Graham (1992) | Highest *vs* lowest quintile | 0.93 | 0.89[a] | 0.81 | 0.86 | 1.07 |
| Willett (1992) | Highest *vs* lowest quintile | | | | | 1.02 |
| Rohan (1993) | Highest *vs* lowest quintile | 0.83 | 0.77 | 0.88 | 0.96 | 0.68 |
| Hunter (1993) | Highest *vs* lowest quintile | 0.80[b,c] | 0.89[d] | 1.03 | 0.90 | |
| Case–control studies | | | | | | |
| Howe (1990)[e] | Highest *vs* lowest quintile | 1.04 | 0.85[b] | 0.69[b] | | 0.85[b] |
| London (1992) | Highest *vs* lowest quintile | 0.9 | 0.6[a] | | 0.4[b] | |
| Levi (1993) | Highest *vs* lowest tertile | 1.0 | 0.4[b] | 0.7 | | |
| Landa (1994) | Highest *vs* lowest tertile | | | 0.40[b] | | 1.38 |
| Baghurst (1994) | Highest *vs* lowest quintile | | | | | 0.48[b] |
| Yuan (1995) | Per unit intake | 0.9 (per 1753 IU) | 0.6[a,b] (per 7269 IU) | 0.3[b] (per 179 mg) | 0.7 (per 30 mg) | 0.4[b] (per 6 g) |
| Freudenheim (1996) | Highest *vs* lowest quartile | | 0.46[b] | 0.53[b] | 0.55[b] | 0.52[b] |

[a]Intake of carotene. [b] Significant association. [c]Preformed vitamin A. [d]Intake of carotenoids with vitamin A activity. [e]Meta-analysis of several case–control studies.

© Cancer Research Campaign 1997 

In our study, supplemental vitamin C intake was not associated with the risk of breast cancer, which is in agreement with results of other epidemiological studies (Graham et al, 1991; Shibata et al, 1992 Hunter et al, 1993). Rohan et al (1993) reported a 40–50% increase in the risk of breast cancer in association with the intake of more than 250 mg day$^{-1}$ vitamin C supplements.

It is suggested that diets rich in PUFAs promote mammary tumour growth by generating lipid peroxy radicals and/or oxygen radicals (Welsch, 1987). Intake of antioxidants would reduce this effect. In our study, the intake of β-carotene or vitamin C was non-significantly negatively associated with breast cancer risk when the intake of PUFAs was high. It should be noted that in a recent pooled analysis of seven cohort studies, including this study, a high intake of PUFAs was not associated with decreased risk of breast cancer (Hunter et al, 1996).

In conclusion, the results of this prospective cohort study provide no evidence of an inverse association between the intake of retinol, β-carotene, vitamin C, vitamin E, dietary fibre, fruit, vegetables and potatoes and the risk of breast cancer. For β-carotene and vitamin C, there was no clear evidence that the intake of PUFAs modifies the effects of β-carotene and vitamin C on breast cancer risk.

### ACKNOWLEDGEMENTS

We are indebted to the participants of this study and further wish to thank the regional cancer registries (IKA, IKL, IKMN, IKN, IKO, IKR, IKST, IKW, IKZ) and PALGA for providing incidence data; Ms S van de Crommert, Ms H Brants, Ms J Nelissen, Ms P Florax, Ms A Pisters and Ms W van Dijk for assistance; Dr A Volovics for statistical advice; and Mr H van Montfort, Mr R Scheimtz, Mr T van Montfort, and Ms M de Leeuw for programming and statistical assistance. This investigation was supported by the Dutch Cancer Society (grant no. TNOV 94–841), the 'Europe against Cancer' programme and the Commodity Board for Vegetables and Fruits.

### REFERENCES

Baghurst PA and Rohan TE (1994) High-fibre diets and reduced risk of breast cancer. *Int J Cancer* **56:** 173–176

Baker RJ (1985) *GLIM 3.77 Reference Manual.* Numerical Algorithms Group: Oxford

Ewertz M and Gill C (1990) Dietary factors and breast-cancer risk in Denmark. *Int J Cancer* **46:** 779–784

Freudenheim JL, Marshall JR, Vena JE, Laughlin R, Brasure JR, Swanson MK, Nemoto T and Graham S (1996) Premenopausal breast cancer risk and intake of vegetables, fruits, and related nutrients. *J Natl Cancer Inst* **88:** 340–348

Goldbohm RA, Van den Brandt PA and Dorant E (1994*a*). Estimation of the coverage of Dutch municipalities by cancer registries and PALGA based on hospital discharge data. *Tijdschr Soc Gezondheidsz* **72:** 80–84

Goldbohm RA, Van den Brandt PA, Brants HA, Van 't Veer P, Al M, Sturmans F and Hermus RJJ (1994*b*) Validation of a dietary questionnaire used in a large-scale prospective cohort study on diet and cancer. *Eur J Clin Nutr* **48:** 253–265

Goldbohm RA, Van 't Veer P, Van den Brandt PA, Van 't Hof MA, Brants HAM, Sturmans F and Hermus RJJ (1995) Reproducibility of a food frequency questionnaire and stability of dietary habits determined from five annually repeated measurements. *Eur J Clin Nutr* **49:** 420–429

Goodwin PJ and Boyd NF (1987) Critical appraisal of the evidence that dietary fat intake is related to breast cancer risk in human. *J Natl Cancer Inst* **79:** 473–485

Graham S, Hellmann R, Marshall J, Freudenheim J, Vena J, Swanson M, Zielezny M, Nemoto T, Stubbe N and Raimondo T (1991) Nutritional epidemiology of postmenopausal breast cancer in western New York. *Am J Epidemiol* **134:** 552–566

Graham S, Zielezny M, Marshall J, Priore R, Freudenheim J, Brasure J, Haughey B, Nasca P and Zdeb M (1992) Diet in the epidemiology of postmenopausal breast cancer in the New York State Cohort. *Am J Epidemiol* **136:** 1327–1337

Howe GR, Hirohata T, Hislop, TG, Iscovich JM, Yuan J-M, Katsouyanni K, Lubin F, Marubini E, Modan B, Rohan T, Toniolo P and Shunzhang Y (1990) Dietary factors and risk of breast cancer: combined analysis of 12 case–control studies. *J Natl Cancer Inst* **82:** 561–569

Howe GR, Friedenreich CM, Jain M and Miller AB (1991) A cohort study of fat intake and risk of breast cancer. *J Natl Cancer Inst* **83:** 336–340

Hunter DJ and Willett WC (1994) Diet, body build, and breast cancer. *Annu Rev Nutr* **14:** 393–418

Hunter DJ, Manson JE, Colditz GA, Stampfer MJ, Rosner B, Hennekens CH, Speizer FE and Willett WC (1993) A prospective study of the intake of vitamins C, E, and A and the risk of breast cancer. *N Engl J Med* **329:** 234–240

Hunter DJ, Spiegelman D, Adami H-O, Beeson L, Van den Brandt PA, Folsom AR, Fraser GE, Goldbohm RA, Graham S, Howe GR, Kushi LH, Marshall JR, McDermott A, Miller AB, Speizer FE, Wolk A, Yaun S-S and Willett WC (1996) Cohort studies of fat intake and the risk of breast cancer – a pooled analysis. *N Engl J Med* **334:** 356–361

Ingram DM, Nottage E and Roberts T (1991) The role of diet in the development of breast cancer: a case–control study of patients with breast cancer, benign epithelial hyperplasia and fybrocystic disease of the breast. *Br J Cancer* **64:** 187–191

Iscovich JM, Iscovich RB, Howe G, Shiboski S and Kaldor JM (1989). A case–control control study of diet and breast cancer in Argentina. *Int J Cancer* **44:** 770–776

Katsouyanni K, Willett WC, Trichopoulos D, Boyle P, Trichopoulou A, Vasilaros S, Papadiamantis J and Macmahon B (1988) Risk of breast cancer among Greek women in relation to nutrient intake. *Cancer* **61:** 181–185

Landa M-C, Frago N and Tres A (1994) Diet and the risk of breast cancer in Spain. *Eur J Cancer Prev* **3:** 313–320

Lee HP, Gourley L, Duffy SW, Esteve J, Lee J and Day NE (1991) Dietary effects on breast-cancer risk in Singapore. *Lancet* **337:** 1197–1200

Levi F, La Vecchia C, Gulie C and Negri E (1993) Dietary factors and breast cancer risk in Vaud, Switzerland. *Nutr Cancer* **19:** 327–335

London SJ, Stein EA, Henderson IC, Stampfer MJ, Wood WC, Remine S, Dmochowski JR, Robert NJ and Willett WC (1992) Carotenoids, retinol, and vitamin E and risk of proliferative benign breast disease and breast cancer. *Cancer Causes Control* **3:** 503–512

*Nevo tabel: Dutch Food Composition Table 1986–1987* (1986) Voorlichtingsbureau voor de Voeding: The Hague.

Paganini-Hill A, Chao A, Ross RK and Henderson BE (1987) Vitamin A, beta-carotene, and the risk of cancer: a prospective study. *J Natl Cancer Inst* **79:** 443–448

Peto R, Doll R, Buckley JD and Sporn MB (1981) Can dietary beta-carotene materially reduce human cancer rates? *Nature* **290:** 201–208

Philips AN and Pocock SJ (1989) Sample size requirements for prospective studies, with examples for coronary heart disease. *J Clin Epidemiol* **42:** 639–648

Richardson S, Gerber M and Cenée S (1991) The role of fat, animal protein and some vitamin consumption in breast cancer: a case–control study in southern France. *Int J Cancer* **48:** 1–9

Rohan TE, Howe GR, Friedenreich CM, Jain M and Miller AB (1993) Dietary fibre, vitamins A, C, and E, and risk of breast cancer: a cohort study. *Cancer Causes Control* **4:** 29–37

Rose DP (1990) Dietary fibre and breast cancer. *Nutr Cancer* **13:** 1–8

Rose DP (1992) Dietary fibre, phytoestrogens, and breast cancer. *Nutrition* **8:** 47–51

Self SG and Prentice RL (1988) Asymptotic distribution theory and efficiency results for case–cohort studies. *Ann Stat* **16:** 64–81

Shibata A, Paganini-Hill A, Ross RK and Henderson BE (1992) Intake of vegetables, fruits, beta-carotene, vitamin C and vitamin supplements and cancer incidence among the elderly: a prospective study. *Br J Cancer* **66:** 673–679

Shultz TD and Howie BJ (1986) *In vitro* binding of steroid hormones by natural and purified fibres. *Nutr Cancer* **8:** 141–147

Steinmetz KA and Potter JD (1991) Vegetables, fruit, and cancer. I. Epidemiology. *Cancer Causes Control* **2:** 325–357

Toniolo P, Riboli E, Protta F, Charrel M and Cappa AP (1989) Calorie-providing nutrients and risk of breast cancer. *J Natl Cancer Inst* **81:** 278–286

Van den Brandt PA, Goldbohm RA, Van 't Veer P, Volovics A, Hermus RJJ and Sturmans F (1990*a*) A large-scale prospective cohort study on diet and cancer in The Netherlands. *J Clin Epidemiol* **43:** 285–295

© Cancer Research Campaign 1997

Van den Brandt PA, Schouten LJ, Goldbohm RA, Dorant E and Hunen PMH (1990*b*) Development of a record linkage protocol for use in the Dutch cancer registry for epidemiological research. *Int J Epidemiol* **19:** 553–558

Van den Brandt PA, Van 't Veer P, Goldbohm RA, Dorant E, Volovics A, Hermus RJJ and Sturmans F (1993) A prospective cohort study on dietary fat and the risk of postmenopausal breast cancer. *Cancer Res* **53:** 75–82

Van den Brandt PA, Goldbohm RA and Van 't Veer P (1995) Alcohol and breast cancer: results from the Netherlands Cohort Study. *Am J Epidemiol* **141:** 907–915

Van 't Veer P, Kolb CM, Verhoef P, Kok FJ, Schouten EG, Hermus RJJ and Sturmans F (1990) Dietary fibre, beta-carotene and breast cancer: results from a case–control study. *Int J Cancer* **45:** 825–828

Welsch CW (1987) Enhancement of mammary tumorigenesis by dietary fat: review of potential mechanisms. *Am J Clin Nutr* **45:** 192–202

Willett WC and Stampfer MJ (1986) Total energy intake: Implications for epidemiologic analyses. *Am J Epidemiol* **124:** 17–27

Willett WC, Stampfer MJ, Colditz GA, Rosner BA, Hennekens CH and Speizer FE (1987) Dietary fat and the risk of breast cancer. *N Engl J Med* **316:** 22–28

Willett WC, Hunter DJ, Stampfer MJ, Colditz G, Manson JE, Spiegelman D, Rosner B, Hennekens CH and Speizer FE (1992) Dietary fat and fibre in relation to risk of breast cancer. *JAMA* **268:** 2037–2044

Yuan J-M, Wang Q-S, Ross RK, Henderson BE and Yu MC (1995) Diet and breast cancer in Shanghai and Tianjin, China. *Br J Cancer* **71:** 1353–1358

Zaridze D, Lifanova Y, Maximovitch D, Day NE and Duffy SW (1991) Diet, alcohol consumption and reproductive factors in a case–control study of breast cancer in Moscow. *Int J Cancer* **48:** 493–501

Ziegler RG (1991) Vegetables, fruits, and carotenoids and the risk of cancer. *Am J Clin Nutr* **53:** 251s–259s

© Cancer Research Campaign 1997